# Enhanced top-down characterization of histone post-translational modifications

**DOI:** 10.1186/gb-2012-13-10-r86

**Published:** 2012-10-03

**Authors:** Zhixin Tian, Nikola Tolić, Rui Zhao, Ronald J Moore, Shawna M Hengel, Errol W Robinson, David L Stenoien, Si Wu, Richard D Smith, Ljiljana Paša-Tolić

**Affiliations:** 1 Environmental Molecular Sciences Laboratory, Pacific Northwest National Laboratory, 902 Battelle Blvd, Richland, WA 99352, USA; 2 Dalian Institute of Chemical Physics, Chinese Academy of Sciences, 457 Zhongshan Road, Dalian, Liaoning 116023, China; 3 Biological Sciences Division, Pacific Northwest National Laboratory, 902 Battelle Blvd, Richland, WA 99352, USA

**Keywords:** Saltless WCX-HILIC, top-down, histone, posttranslational modification

## Abstract

Post-translational modifications (PTMs) of core histones work synergistically to fine tune chromatin structure and function, generating a so-called histone code that can be interpreted by a variety of chromatin interacting proteins. We report a novel online two-dimensional liquid chromatography-tandem mass spectrometry (2D LC-MS/MS) platform for high-throughput and sensitive characterization of histone PTMs at the intact protein level. The platform enables unambiguous identification of 708 histone isoforms from a single 2D LC-MS/MS analysis of 7.5 µg purified core histones. The throughput and sensitivity of comprehensive histone modification characterization is dramatically improved compared with more traditional platforms.

## Background

Histones are important chromatin proteins that act as spools to package and order DNA into structural and manageable chromosomes. Core histones are modified by multiple post-translational modifications (PTMs) such as lysine acetylation, lysine or arginine methylation, and serine or threonine phosphorylation, among others. These PTMs generate a 'histone code' [1] that is implicated in chromatin-related cellular processes [2] including transcription [3], replication [4], repair [5], and alternative splicing [6].

Although core histones comprise only four families (H4, H2B, H2A, and H3), each family has thousands of potential isoforms generated by different combinations of PTMs and protein sequence variation. Traditional antibody-based methods target specific isoforms, typically analyzing one PTM at a time, which makes it virtually impossible to measure combinatorial modifications occurring within the same histone molecule. Recently, high-throughput bottom-up [7] and middle-down [8] proteomic methods demonstrated potential for global characterization of PTMs on histone tails. However, these methods are ill-suited for characterizing multiple PTMs dispersed along the entire protein sequence that have been previously discovered to have significant participation in chromatin regulation [2,9-11].

Top-down proteomic and high-throughput approaches are clearly required to identify and quantify the modulation of multiple intra-molecular histone modifications that synergistically regulate histone functions. Recently, a global top-down study demonstrated the feasibility of intact protein analysis for this purpose by identifying more than 300 histone isoforms using extensive fractionation and customized bioinformatics for global proteome characterization [12]. In histone-focused studies, top-down approaches using an offline two-dimensional liquid chromatography (2D LC) separation and Fourier transform mass spectrometry (FTMS) characterized 34 H4 isoforms from approximately 150 μg of purified H4 protein [13]. However, this study demanded several separations and purification steps for MS compatible samples, requiring a large quantity of starting material and limiting throughput. Clearly, this offline approach is labor-intensive and time-consuming, and requires relatively large sample sizes preventing analysis of biological samples of limited availability such as tumor specimens.

Traditionally, a mobile phase with high-concentration salt in weak cation exchange - hydrophilic interaction LC (WCX-HILIC) has been utilized to separate acetylated [14] and methylated [15] histone isoforms. However, the presence of high concentration salts (for example, NaClO_4_) in the elution buffer leads to ionization suppression and is, therefore, incompatible with modern electrospray ionization (ESI) interfaces typically used for high-throughput online analysis of protein mixtures. Recently, Young *et al*. successfully developed an alternative 'saltless' pH gradient WCX-HILIC for online middle-down proteomic analysis of human histone H3.2, which enabled an approximately 100-fold reduction in sample requirements and analysis time [8].Similarly, in this study we utilized a salt-free pH-gradient WCX-HILIC [8] as the second dimension for separating differentially acetylated/methylated intact protein isoforms within each histone family (H4, H2B, H2A, H3). We combined this separation with online reversed-phase LC (RPLC) in the first dimension to separate histone families and FTMS to enhance MS characterization of intact histones.

In this article, we report a novel high-throughput and high-sensitivity platform for comprehensive characterization of combinatorial histone PTMs at the intact protein level. The novelty stems from use of a metal-free online 2D LC that is coupled with high-performance FTMS. The platform enabled unambiguous identification of 708 histone isoforms from a single analysis of 7.5 μg HeLa core histones.

## Results and discussion

### Analyses of core histones

**Figure 1:**
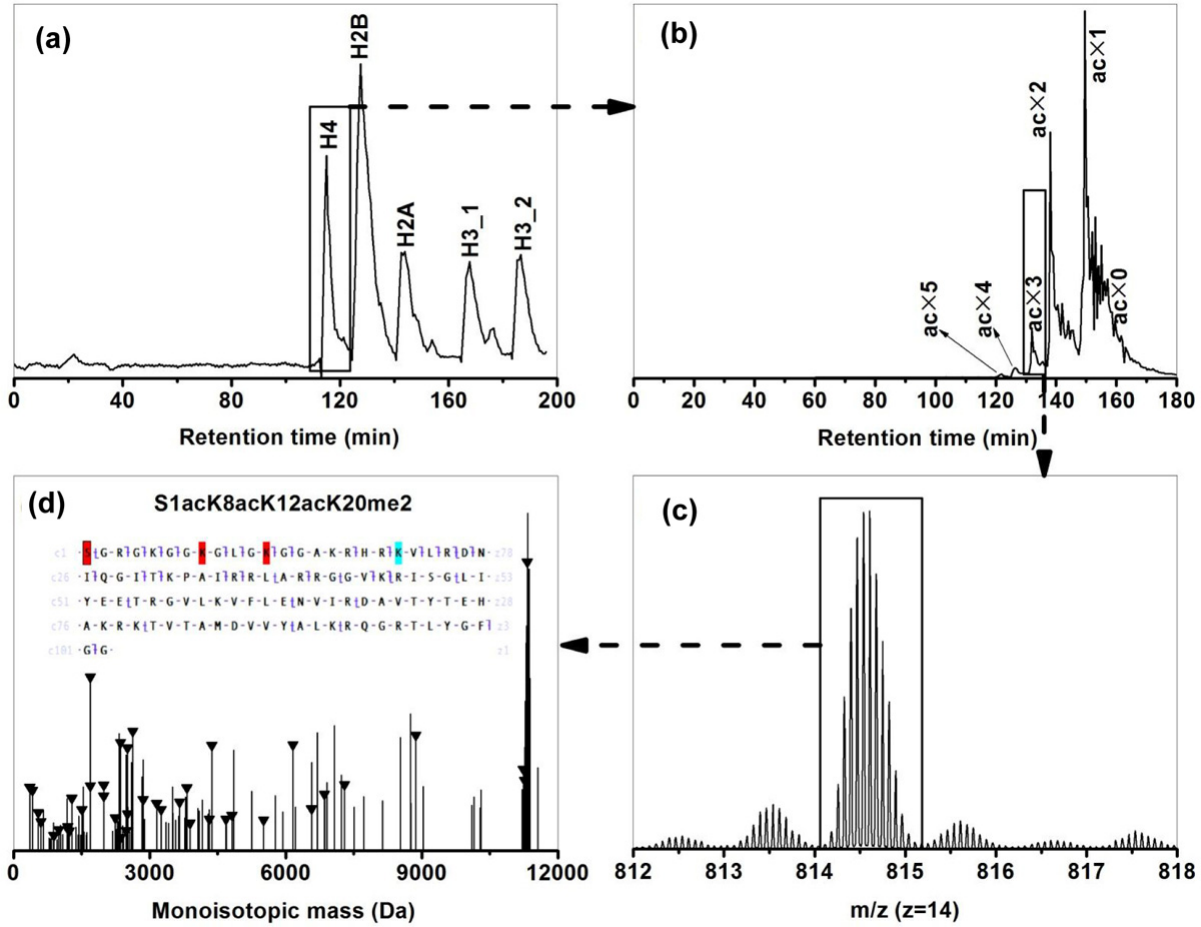
**Overall experimental workflow as illustrated by the identification of H4 (P62805) isoform S1acK8acK12acK20me2**. **(a) **UV chromatogram from first dimension RPLC separation of 7.5 μg of HeLa core histone mixture. **(b) **MS-only total ion current (TIC) chromatogram from WCX-HILIC-MS/MS analysis of H4 fraction from the first dimension. **(c) **Representative mass spectrum (only charge state 13 shown) at retention time 136.54 min from WCX-HILIC-MS/MS analysis of H4 fraction from the first dimension. **(d) **Representative deconvoluted CID spectrum for precursor ion *m*/*z *877.12 with matching fragments marked with 'triangles'. The inset is the matching fragment mapping on the protein amino acid sequence with PTMs color-coded. CID, collision induced dissociation; PTMS, post-translation modifications; RPLC, reversed phase liquid chromatography; WCX-HILIC-MS/MS, weak cation exchange-hydrophilic interaction liquid chromatography-tandem mass spectrometry.

A UV chromatogram from the first dimension RPLC analysis (Figure 1a) demonstrates baseline separation of core histones into individual family members H4, H2B, H2A, and H3 that appear in increasing order of hydrophobicity. Isoforms in each family elute together in a single chromatographic peak with the exception of H3 which elutes in two distinct peaks. The elution order is in accord with the increasing average family molecular weight, that is, 11,352.5, 13,757.1, 14,019.9, and 15,350.8 Da for H4, H2B, H2A, and H3, respectively, and follows increasing protein hydrophobicity as expected for reverse phase separations. In the case of H3, isoforms eluting in the second peak are slightly more hydrophobic as they contain one to three extra methylations on average. Separation of core histones into individual families is advantageous for further downstream analyses including separation, fragmentation, and identification.

**Figure 2:**
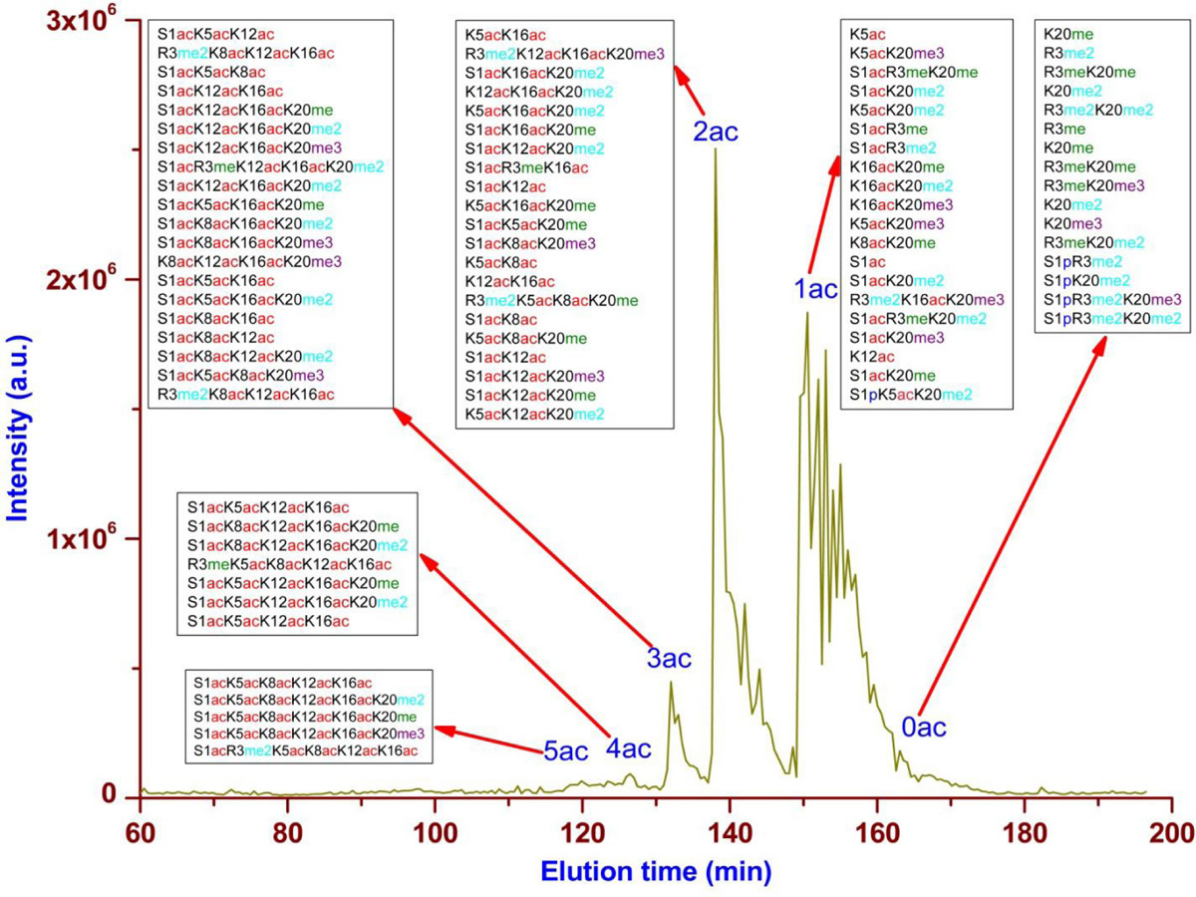
**Separation of H4 isoforms according to degree of acetylation using WCX-HILIC**. Isoforms with more acetylation carry less positive charges and, thus, have weaker electrostatic interaction with negative function groups (carboxylate for PolyC AT A) on the stationary phase and elute earlier.

In the second dimension WCX-HILIC separation, isoforms within each histone family are separated primarily based on the degree of acetylation, as identified by the intact mass and tandem mass spectra (Figure 1b, c and 1d). The number of positive amine charges decreases as the degree of acetylation increases, which causes the isoform to elute earlier due to the weaker electrostatic interaction with the stationary phase (poly-aspartic acid). In addition to ionic interactions, hydrophilic interactions between analyte and stationary phase become significant because a high organic mobile phase (70% acetonitrile (ACN)) is used [16], resulting in a secondary separation related to the total number of methylations within each differentially acetylated subgroup. An MS-only base-peak chromatogram obtained for the H4 fraction in the second dimension displays isoforms containing up to five acetylation groups chromatographically resolved with partial resolution of methylation groups (Figure 2). Isoforms with up to two and three acetylation groups were resolved within H2 and H3 families, respectively (data not shown). A total of 708 histone isoforms were identified across the four core histone families from 7.5 µg of sample. Specifically, after application of the filtering criteria described above, a *P *score less than 1E-4, and false discovery rate (FDR) less than 1%, 105, 110, 77, and 416 isoforms were identified for H4, H2B, H2A, and H3, respectively, using the 2D RP-WCX-HILIC LC-MS/MS platform (Table 1, more detailed information is provided in Additional files 1 to 5). A key advantage of the metal-free set-up is the enhanced ability to detect phosphorylated isoforms, which comprised 14% of the total isoforms identified. Isoforms with up to four distinct phosphorylation sites, for example, A1acT3**p**R8me2K9acS10**p**T11**p**K14acK18 acK23acK27acS28**p**K36ac (H31T, Q16695), were confidently identified.

**Table 1:** Histone isoforms identified from 2D RP-WCX-HILIC LC-MS/MS analysis of 7

	H4	H2B	H2A	H3	Total
RP-WCX-HILIC	105 (10)	110 (19)	77 (28)	416 (69)	708 (126)
RPLC	36 (1)	21 (0)	58 (8)	12 (1)	127 (10)
WCX-HILIC	27 (3)	64 (9)	26 (4)	18 (3)	136 (19)

The total number of isoforms for each core histone family member are shown. The numbers in parentheses represent phosphorylated isoforms. RPLC, reversed phase liquid chromatography; WCX-HILIC, weak cation exchange-hydrophilic interaction liquid chromatography.

### Comparison of CID versus ETD

**Figure 3:**
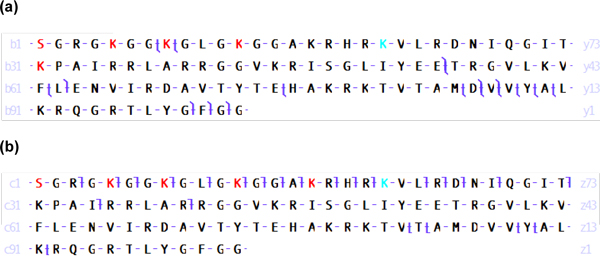
**Comparison of ETD versis CID**. Fragment maps of H4_S1acK5acK8acK12acK16acK20me2 from CID (a) and ETD (b) for the parent ion with m/z = 820.61 (z = 16) eluting at 60.88 min are shown. CID, collision induced dissociation ETD, electron transfer dissociation.

In this study, collision induced dissociation (CID) and electron transfer dissociation (ETD) were used in parallel, that is, ETD was performed on the same precursor ion immediately following CID. Using ProSightPC for identifications, if CID and ETD each produced identification for the same precursor ion, the spectrum with the best *P *score was reported. Using the identification criteria adopted in this study, 158 and 550 histone isoforms were identified using CID and ETD, respectively, in the 2D RP-WCX-HILIC LC-MS/MS analysis of HeLa core histones (Table 2). Overall, ETD provided two to three times more identifications compared to CID. We have noted that fragmentation using ETD typically results in consecutive fragmentation along the N-terminal region with minimal internal fragmentation. Similarly, fragmentation using CID typically results in fewer observed total fragments but included fragments throughout the histone backbone. This distinct behavior between CID and ETD was illustrated in the fragmentation and identification of H4_S1acK5acK8acK12acK16acK20me2 (Figure 3). Depending on which sites are modified, each method has the potential to outperform the other. A comprehensive study of CID, ETD, and high-energy collision dissociation (HCD) efficacy for the identification of histone isoforms has been performed, but is out of the scope of this study and will be reported elsewhere.

**Table 2:** Comparison of CID and ETD for histone isoform identification using 2D RP-WCX-HILIC LC-MS/MS analysis.

	H4	H2B	H2A	H3	Total
Subtotal	105 (10)	110 (19)	77 (28)	416 (69)	708 (126)
CID	48 (5)	57 (17)	9 (4)	44 (8)	158 (34)
ETD	57 (5)	53 (2)	68 (24)	372 (61)	550 (92)

The total numbers of isoforms identified by either CID or ETD are shown with phosphorylated isoforms in parentheses. CID, collision induced dissociation; ETD, electron transfer dissociation; 2D RP-WCX-HILIC LC-MS/MS, two dimensional reverse phase**-**weak cation exchange-hydrophilic interaction liquid chromatography-tandem mass spectrometry.

### Comparison of 2D versus 1D separation

**Figure 4:**
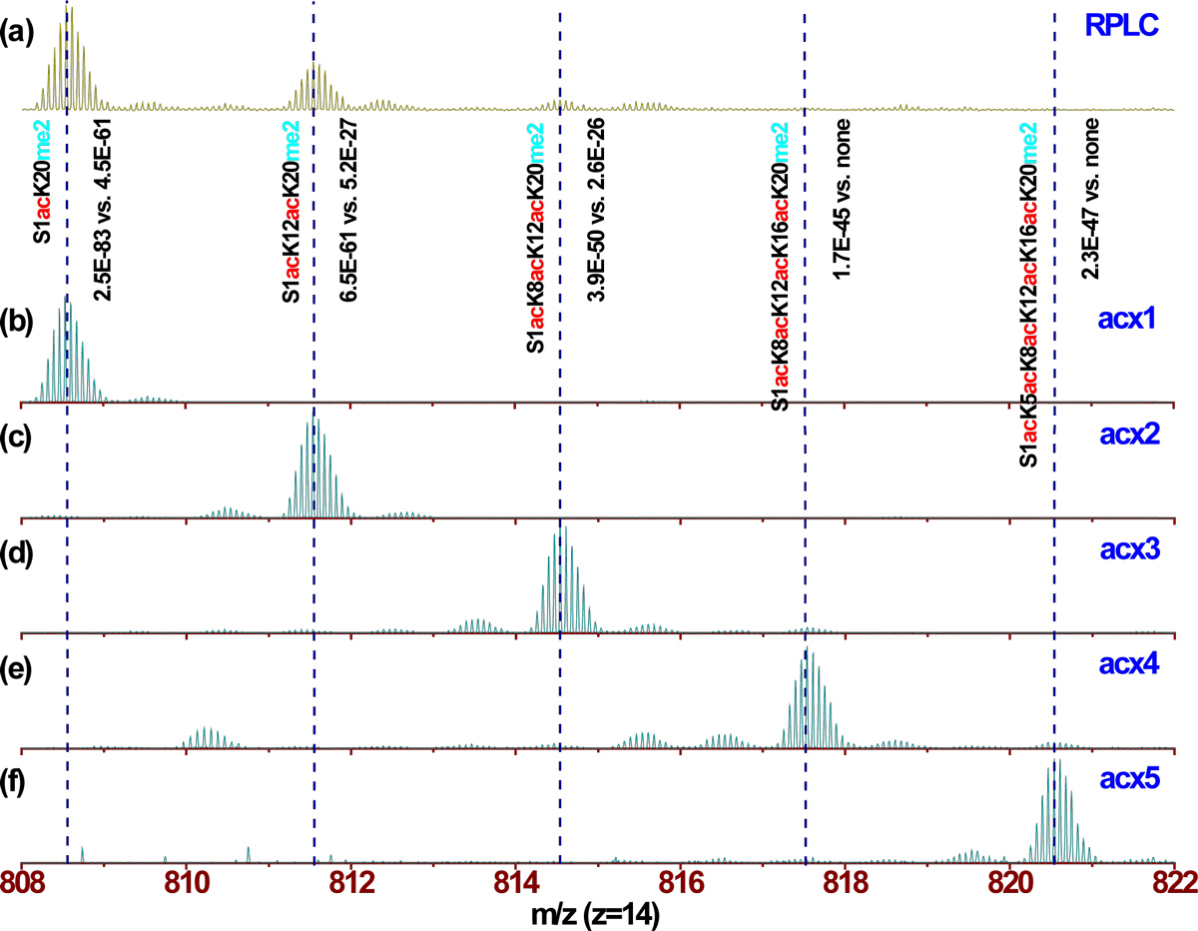
**Increase of detection dynamic range with 2D separation**. Representative mass spectra (charge state 14+) for chromatographic peaks shown in Figure 1. **(a) **Isoforms of H4 observed from RPLC separation of HeLa core histones; **(b to f) **isoforms of H4 observed from 2D RP/WCX-HILIC separation of histone H4. Isoforms identified from the most abundant peaks as marked with the dotted lines together with *P *scores (top, RPLC; bottom: WCX-HILIC) are noted above (b). RPLC, reversed phase liquid chromatography; WCX-HILIC, weak cation exchange-hydrophilic interaction liquid chromatography.

In comparison, LC-MS analysis of core histones using either RPLC or WCX-HILIC alone identified 127 and 135 histone isoforms, respectively (See Additional files 6 and 7). The 2D separation identified five to six times more isoforms in total than either of the 1D separations in this study (Table 1). The largest difference was observed for the H3 family, which is the most complex family and thus benefits most from an additional dimension of separation by increasing dynamic range and MS sampling time. For example, in the RPLC only analysis, histone H4 isoforms S1acK20me2, S1acK12acK20me2, and S1acK8acK12acK20me2 are observed to co-elute with S1acK20me2 being the most abundant ion (Figure 4). With an additional dimension of separation using WCX-HILIC, not only are S1acK12acK20me2 and S1acK8acK12acK20me2 chromatographically separated from S1acK20me2, but also represent the most abundant peak in each respective mass spectrum. For the three example isoforms identified in both RPLC and 2D analyses described above, the *P *scores from the 2D analysis improved by 22, 34, and 24 orders of magnitude compared to those from the 1D RPLC analysis. Additionally low abundant isoforms S1acK8acK12acK16acK10me2 and S1acK5acK8acK12acK16acK10me2, which are not observed in the RPLC 1D analysis, are newly separated chromatographically and elute as the most abundant peaks in the respective mass spectra allowing for confident identification. As protein forms in MS spectra are selected for fragmentation in order of decreasing intensity, improved separation allows for better peak detection and a greater opportunity for selection of lower abundance species for dissociation.

### Comparison of this online top-down study with offline top-down, bottom-up, and middle-down studies

**Figure 5:**
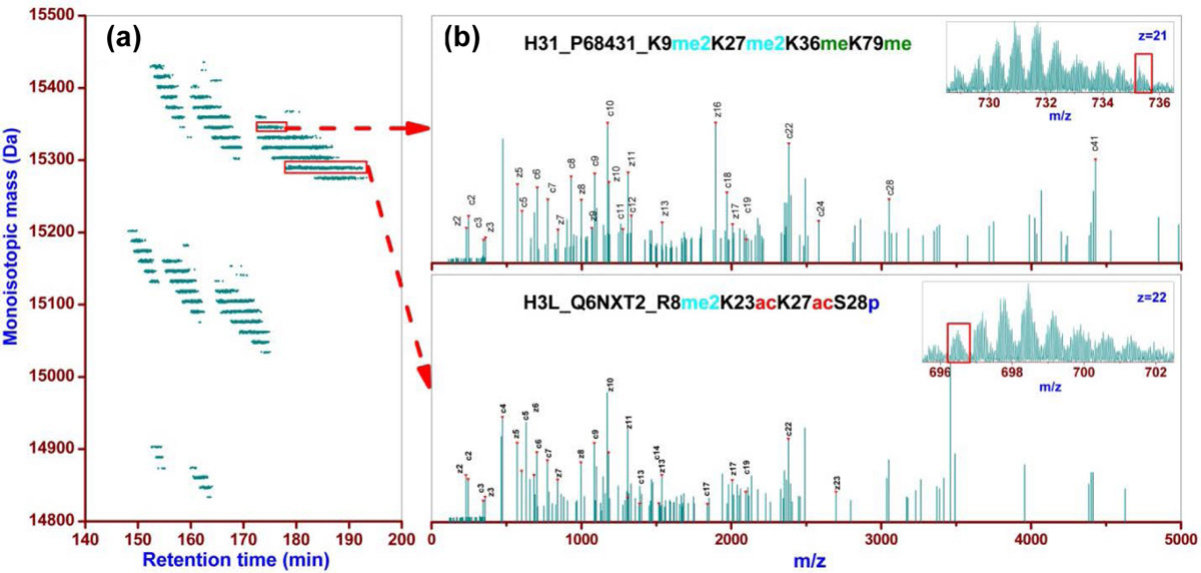
**Representative isoforms with PTMs beyond the N-terminal tail and with phosphorylation**. **(a) **Intact H3 isoforms observed from second dimension WCX-HILIC analysis of H31 fraction as shown in Figure 1 (a). **(b) **Example tandem mass spectra for an isoform displaying modification beyond the histone tail (top) and a phosphorylated isoform (bottom). Insets show the corresponding parent ion spectra with precursor ions selected for fragmentation boxed. PTM, post-translation modification; WCX-HILIC, weak cation exchange-hydrophilic interaction liquid chromatography.

Most recently, the use of top-down proteomics coupled with fractionation demonstrated the complexity of histone isoforms within a whole proteome study [12]; here, the results expand upon the number of identifications providing a more detailed report of histone isoforms. With the 'saltless' pH gradient used in this study, WCX-HILIC can be coupled directly with ESI without the previous restrictions of desalting or sample dilution, increasing the throughput and sensitivity. In comparison with previous offline top-down analysis of H4 [13], this online study identified approximately three times the number of isoforms from approximately 100-fold less sample. Similarly, our 2D LC FTMS platform increased the number of previously reported H4 identifications using bottom-up proteomics [17] and identified combinatorial modifications that are unattainable using a bottom-up approach. To date, no comprehensive characterization of H2B, H2A, and H3 at the intact protein level has been reported to the best of our knowledge. Among the 416 identified H3 isoforms (Table 1), 98 (24%) contain single or multiple modifications beyond the histone tail (that is, the first 50 amino acids explored in middle down studies). These modifications are potentially biologically relevant. For example, K9me2K27me2K36meK79me (H31, P68431), identified with *P *score of 3.8×10^-33 ^(Figure 5), displays methylation on K79, which has been related to epigenetic silencing and DNA repair [18]. Other potentially interesting modification sites identified in this study include phosphorylation on S47 and T51 of H4, whereas previous offline top-down [13] and middle-down [17] approaches have been limited to detect modifications up to K20 within the N-terminal tail. Conflicting histone marks such as PTMs predicted to activate or repress transcription can occur simultaneously within the same histone isoform, with the potential to result in greater selectivity in the epigenetic regulation of specific target genes, further highlighting the need for top-down analysis of histone modifications.

While results both presented here and recently published by Tran *et al*. [12] are promising in both the field of top-down proteomics and histone analysis, the number of identifications may be influenced by the lack of a histone specific top-down bioinformatics platform. While sequence tags and precursor accurate mass are sufficient for traditional top-down proteomic analyses, histone analysis is complicated by several factors. Such complications include: modification-positional isomers; the small delta mass between acetylation and trimethylation; unknown modifications and those associated with sample processing (that is, oxidation), which could potentially lead to misassignment when searching against databases restricted to known modifications; co-fragmentation of multiple isoforms due to crowding of isotopic distributions in the *m/z *space; and correct deisotoping. While limiting the search space to previously defined modifications may be required using current tools to complete searches in a realistic time frame (that is, a few days), ultimately, previously unidentified modification sites and forms will not be identified, which brings to light the need for a different type of bioinformatics platform specific to histone analysis. Some of these concerns are addressed by DiMaggio *et al*. [19]; however, scaling this middle out tool or others available for the more complicated top-down realm has yet to be achieved. Specific scoring functions are required for ranking the confidence/probability of deisotoped intact mass, site localization of each modification, and protein sequence identification. Additionally, presumably many of the unidentified spectra contain enough fragment ions to assign the correct protein sequence (that is, protein identification), but not sufficient ions to confidently localize PTM site(s) (that is, protein isoform characterization), which is required for comprehensive histone analysis. This gap between protein identification versus characterization will become a larger issue as the popularity of top-down analysis increases, and will hopefully drive the development of a tailored suit of bioinformatics tools for these types of analyses. Co-current optimization of MS technologies/fragmentation methods for histone analysis and bioinformatics platforms that provide confident identifications are needed for comprehensive identifications.

## Conclusions

In conclusion, online 2D separation using RP followed by HILIC chromatography allows for the detection and identification of more than seven hundred histone isoforms in a top-down fashion. These results highlight the complexity of histones in general, and demonstrate that modifications that may be important components of the histone code extend well beyond the histone tail region. In general, we envision the metal-free RPLC-WCX/HILIC-FTMS platform being used in a broad range of applications, not only for epigenetic studies of histones, but also for the study of combinatorial PTMs that regulate other classes of proteins.

## Materials and methods

The metal-free 2D LC system used in this study is configured as previously reported [20], except that the system has been further optimized by exchanging the order of the separations and new buffers were developed as described below. A schematic diagram of the new system is shown in Additional file 8. MS grade solvents were obtained from Thermo Fisher Scientific (Waltham, MA, USA).

### First dimension RPLC-UV analysis of HeLa core histone mixture

A total of 7.5 μg purified HeLa core histones (Active Motif, Carlsbad, CA, USA) were separated in the first dimension using a Jupiter C5 (5 μm particles, 300 Å pore size) (Phenomenex, Torrance, CA, USA) column (600 mm × 200 μm i.d.) packed in-house. The separation was carried out under constant pressure at 4,000 psi using two Model 100 DM 10,000 psi syringe pumps (with Series D Pump Controller) (ISCO, Lincoln, NE, USA). Mobile phase A consisted of 20% ACN aqueous solution with 5% isopropanol alcohol (IPA) and 0.6% formic acid (FA); mobile phase B consisted of 45% ACN, 45% IPA, and 0.6% FA. The gradient was generated by adding mobile phase B (4,000 psi) to a stirred mixer (volume 2.5 mL equilibrated with 100% mobile phase A at time zero), where an appropriate split flow rate was controlled by the combination of a packed column together with 15 μm i.d. capillary, with an approximate flow of 10 µL/min. Protein elution was monitored online at 214 nm with a SPECTRA100 UV detector (Thermo Separation Products, Waltham, MA, USA). Fractions of interest were collected using two Cheminert column selector systems (VICI, Houston, TX, USA). Once a fraction was collected in one column selector system from the first dimension, the fractionation was switched to the other column selector system and further separation of the first collected fraction in the second dimension ensued.

### Second dimension WCX-HILIC-MS/MS analyses of individual histone families

Each histone family fraction was further separated in the second dimension by WCX-HILIC using a PolyCAT A (5 μm particles, 1000 Å pore size) (PloyLC, Columbia, MD, USA) column (50 cm × 100 μm i.d.) packed in house. The separation was carried out with equipment identical to the first dimension mentioned above except for using 70% ACN aqueous solution with 1.0% FA for Mobile phase A and 70% ACN and 8% FA for Mobile phase B. A Cheminert ten-port Nanovolume injection valve (VICI) was used to house two capillary columns, enabling separation and concurrent loading/equilibration between the two columns to increase the throughput of the second dimension. The isolated histone fraction was first loaded onto a solid phase extraction (SPE) column (150 μm i.d. × 5 cm, HILIC stationary phase described above) using Mobile phase A from the second dimension. Once the loading process of one fraction was finished Mobile phase B from the second dimension was added to the mixing vessel to separate the loaded protein and ESI high-resolution MS and MS/MS acquisitions in a LTQ Orbitrap Velos (ThermoFisher Scientific, Waltham, MA) were initiated. ESI voltage was applied by connecting the end of the LC column to a 20 µm i.d. chemically etched capillary emitter with a PEEK union while a voltage was applied through a metal union coupled in the split/purge line out of the analyte path. All acquisitions were performed by the Orbitrap with nominal resolving power of 60,000 (*m/z *= 400). FTMS MS and MS^n ^automatic gain control (AGC) target values were 1E6 and 3E5, respectively. The number of micro scans for both MS and MS^n ^was three. Fragmentation of precursor ions, isolated with a 1.5 *m/z *window, was performed by alternating CID (normalized collision energy 35%, 30 ms) and ETD (reaction time 25 ms) for the same precursor ion. Dynamic exclusion was implemented with exclusion duration of 900 s and an exclusion list size of 150. MS/MS was only performed on species with charge states greater than four.

One-dimensional analyses of HeLa core histones using RPLC or WCX-HILIC under the mass spectrometric conditions above were also carried out for the purpose of comparison with two-dimensional analysis.

Raw MS data for both the one- and two- dimensional datasets were deposited in the PeptideAtlas repository [21]. The URLs to access these datasets are [22-27].

### Protein identification

Protein isoforms as well as PTMs were identified by searching each RAW dataset against an annotated top-down human database (official_human_TD, 117,059 basic sequences, and 7,563,274 protein forms) using ProSightPC 2.0 (Thermo Scientific, Waltham, MA). An embedded Thrash algorithm was chosen to deconvolute both precursor and fragment ions. The minimum signal-to-noise (S/N), minimum reliability (RL), maximum charge and maximum mass were set to 1.0, 0.9, 40, and 25 kDa, respectively. Individual spectra were searched in absolute mass mode if a minimum of six fragments and minimum intact mass of 5,000 Da were observed, and the fragment mass tolerance was set at 10 ppm. Dynamic PTMs considered include methylation (mono-, di-, tri-), acetylation, and phosphorylation. Histone identifications were filtered by requiring the 'Number of Best Hits' to be one (globally unique ID). FDR was evaluated using reversed database search with the same filtering criteria, where FDR = 100*N_reverse_/N_forward_. When a *P *score cutoff of 1E-4 was chosen, FDR is less than 1% (Table 3).

**Table 3:** Change of FDR with different *P *score cutoff.

P score cutoff	1.0E-2	1.0E-3	1.0E-4	1.0E-5
IDs_Reversed	21	15	8	4
IDs_Forward	708	707	697	684
FDR (/%)	3.0	2.1	1.1	0.6

Shown are the effects of altering P score cutoffs on the false discovery rate (FDR).

## Abbreviations

2D LC: two-dimensional liquid chromatography; ACN: acetonitrile; CID: collision induced dissociation; ESI: electrospray ionization; ETD: electron transfer dissociation; FA: formic acid; FDR: false discovery rate; FTMS: Fourier transform mass spectrometry; IPA: isopropanol alcohol; kDa: kiloDaltons; PTMs: post-translational modifications; RPLC: reversed-phase LC; SPE,solid phase extraction; WCX-HILIC: weak cation exchange - hydrophilic interaction liquid chromatography.

## Competing interests

The authors declare that they have no competing interests.

## Authors' contributions

ZT, NT and RZ carried out the experiments and did the data analysis. RJM, SMH, EWR, DLS and SW helped either with the sample preparation or the LC-MS analyses. ZT, RDS and LPT conceived the study and participated in the study design. ZT drafted the manuscript. All authors read, revised and approved the final manuscript for publication.

## Supplementary Material

Additional file 1 **Additional information regarding histone H4 tandem MS results**. Data are contained in five sheets in a Microsoft Excel file format. Sheet one contains a collection of information from the data analysis software. Sheet two contains protein isoform information specific to ETD fragmentation. Sheet three contains protein isoform information specific to CID fragmentation. Sheet four summarizes protein isoform information common to both CID and ETD. Sheet five summarizes ETD and CID fragment ions. CID, collision induced dissociation; ETD, electron transfer dissociation; MS, mass spectrometry. Click here for file

Additional file 2 **Additional information regarding histone 2B tandem MS results**. Data are contained in three sheets in a Microsoft Excel file format. Sheet one contains a summary of information from the data analysis software. Sheet two contains protein isoform information specific to CID fragmentation. Sheet three contains protein isoform information specific to ETD fragmentation. CID, collision induced dissociation; ETD, electron transfer dissociation; MS, mass spectrometry. Click here for file

Additional file 3 **Additional information regarding histone 2A tandem MS results**. Data are contained in three sheets in a Microsoft Excel file format. Sheet one contains a summary of information from the data analysis software. Sheet two contains protein isoform information specific to ETD fragmentation. Sheet three contains protein isoform information specific to CID fragmentation. CID, collision induced dissociation; ETD, electron transfer dissociation; MS, mass spectrometry. Click here for file

Additional file 4 **Additional information regarding histone 3 first fraction tandem MS results**. Data are contained in three sheets in a Microsoft Excel file format. Sheet one contains a summary of information from the data analysis software. Sheet two contains protein isoform information specific to CID fragmentation. Sheet three contains protein isoform information specific to ETD fragmentation. CID, collision induced dissociation; ETD, electron transfer dissociation; MS, mass spectrometry. Click here for file

Additional file 5 **Additional information regarding histone 3 second fraction tandem MS results**. Data are contained in three sheets in a Microsoft Excel file format. Sheet one contains a summary of information from the data analysis software. Sheet two contains protein isoform information specific to ETD fragmentation. Sheet three contains protein isoform information specific to CID fragmentation. CID, collision induced dissociation; ETD, electron transfer dissociation; MS, mass spectrometry. Click here for file

Additional file 6 **Additional information regarding histones from a single RPLC separation**. Data are contained in a Microsoft Excel file format and present a summary of information from the data analysis software for the results from a single dimension RPLC separation. RPLC, reversed phase liquid chromatography. Click here for file

Additional file 7 **Additional information regarding histones from a single WCX-HILIC separation**. Data are contained in a Microsoft Excel file format and present a summary of information from the data analysis software for the results from a single dimension WCX-HILIC separation. WCX-HILIC, weak cation exchange-hydrophilic interaction liquid chromatography. Click here for file

Additional file 8 **Schematic diagram of the online metal-free RPLC/WCX-HILIC system**. Details of the experimental set-up used to perform the described HPLC separations, including valve and flow path details. HPLC, high performance liquid chromatography; RPLC/WCX-HILIC, reversed phase liquid chromatography-weak cation exchange-hydrophilic interaction liquid chromatography. Click here for file
