# Supplementary material for: Enhanced top-down characterization of histone post-translational modifications
Source: Genome Biol. 2012 Oct 3;13(10):R86. doi: 10.1186/gb-2012-13-10-r86 (PMC3491414; doi:10.1186/gb-2012-13-10-r86)
Supplement: Additional file 8 — Schematic diagram of the online metal-free RPLC/WCX-HILIC system. Details of the experimental set-up used to perform the described HPLC separations, including valve and flow path details. HPLC, high performance liquid chromatography; RPLC/WCX-HILIC, reversed phase liquid chromatography-weak cation exchange-hydrophilic interaction liquid chromatography. [file gb-2012-13-10-r86-S8.docx]

**Figure S1.** Schematic diagram of the online metal-free RPLC/WCX-HILIC system. Two Cheminert 5000 psi 6-port metal-free Nanovolume injection valves (V1 and V2) were used in the first dimension to facilitate sample injection to the sample loop, loading to the SPE column (SPE1), and RPLC separation (column 1). The eluate from the first dimension was monitored with an online UV detector before it was directed into the storage and then the second dimension separation using fractionation valves (V3, V4, V5, and V6). In the second dimension, a same type valve (but 10-port instead of 6-port) (V7) was used to control loading of the first dimension fraction onto the SPE columns (SPE2 and SPE3), separation with two WCX-HILIC columns (columns 2 and 3), and equilibration of the WCX-HILIC columns. Mixers 1 and 2, initially filled with Buffer As, were used to generate elution gradient by adding Buffer Bs with a certain split flow rate. Two metal unions were coupled in the split/purge line to apply the high voltage (marked as HV) for ESI. V, valve; *W*, waste; MS, mass spectrometer.
